# Epigenetic acquisition of inducibility of type III cytotoxicity in *P. aeruginosa*

**DOI:** 10.1186/1471-2105-7-272

**Published:** 2006-05-30

**Authors:** Didier Filopon, Annabelle Mérieau, Gilles Bernot, Jean-Paul Comet, Rozenne LeBerre, Benoit Guery, Benoit Polack, Janine Guespin-Michel

**Affiliations:** 1 GREPI EA 2938 CHU de Grenoble, BP 217, 38043 Grenoble cedex 9, France; 2 Laboratoire de microbiologie du froid, EA 2123, Université de Rouen, F-76 821 Mt St Aignan, France; 3 LaMI, CNRS UMR 8042, Université d'Évry-Val-d'Essonne, Boulevard François Mitterrand, 91025 Évry, France; 4 EA 2689, Faculté de Médecine, Pole Recherche, CHRU de Lille, 1 Place de Verdun, 59045 Lille, France

## Abstract

**Background:**

*Pseudomonas aeruginosa*, an opportunistic pathogen, is often encountered in chronic lung diseases such as cystic fibrosis or chronic obstructive pneumonia, as well as acute settings like mechanical ventilation acquired pneumonia or neutropenic patients. It is a major cause of mortality and morbidity in these diseases. In lungs, *P. aeruginosa *settles in a biofilm mode of growth with the secretion of exopolysaccharides in which it is encapsulated, enhancing its antibiotic resistance and contributing to the respiratory deficiency of patients. However, bacteria must first multiply to a high density and display a cytotoxic phenotype to avoid the host's defences. A virulence determinant implicated in this step of infection is the type III secretion system (TTSS), allowing toxin injection directly into host cells. At the beginning of the infection, most strains isolated from patients' lungs possess an inducible TTSS allowing toxins injection or secretion upon *in vivo *or *in vitro *activation signals. As the infection persists most of the bacteria permanently loose this capacity, although no mutations have been evidenced. We name "non inducible" this phenotype. As suggested by the presence of a positive feedback circuit in the regulatory network controlling TTSS expression, it may be due to an epigenetic switch allowing heritable phenotypic modifications without genotype's mutations.

**Results:**

Using the generalised logical method, we designed a minimal model of the TTSS regulatory network that could support the epigenetic hypothesis, and studied its dynamics which helped to define a discriminating experimental scenario sufficient to validate the epigenetic hypothesis. A mathematical framework based on formal methods from computer science allowed a rigorous validation and certification of parameters of this model leading to epigenetic behaviour. Then, we demonstrated that a non inducible strain of *P. aeruginosa *can stably acquire the capacity to be induced by calcium depletion for the TTSS after a short pulse of a regulatory protein. Finally, the increased cytotoxicity of a strain after this epigenetic switch was demonstrated *in vivo *in an acute pulmonary infection model.

**Conclusion:**

These results may offer new perspectives for therapeutic strategies to prevent lethal infections by *P. aeruginosa *by reverting the epigenetic inducibility of type III cytotoxicity.

## Background

**Figure 1:**
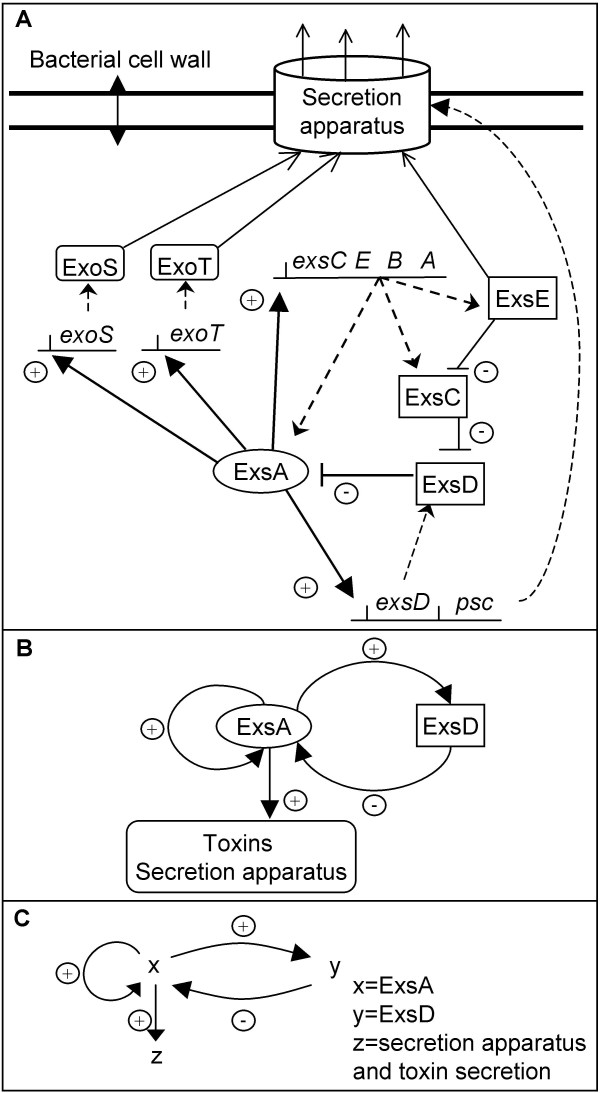
**Models of the Regulation of the TTSS**. (A) The regulatory network of the type III secretion system. Regulators encoded outside of the ExsA regulon are not shown. Filled arrows and dashed lines represent positive or negative interactions respectively, dotted lines stand for transcription and translation, opened arrows represent the secretion of proteins. (B) The molecular sub-network drawn here only shows interactions involved in feedback circuits: autoregulation of *exsA*, activation by ExsA of the operons involved in the cytotoxic response, and the inhibition of ExsA by ExsD. Induction of secretion and expression of cytotoxicity by the target, or by calcium depletion, is considered constant and is therefore not indicated. Other regulations of the *exsA *gene are not represented. (C) The minimal regulatory graph extracted from the molecular graph. The three variables are x = ExsA, y = ExsD (the ExsA inhibitor), and z = type III secretory apparatus and toxin secretion. The four arrows represent autoregulation of the *exsA *gene (x→x), transcriptional activation of the *exsD *gene by protein ExsA (x→y), transcriptional activation of the genes involved in type III secretory system (x→z), and inhibition of ExsA by ExsD (y→x).

*Pseudomonas aeruginosa *is a Gram-negative opportunistic pathogen associated with sepsis in burned, neutropenic, and intensive care patients as well as with severe chronic lung injury in cystic fibrosis and chronic obstrusive pneumonia disease[1]. Cytotoxic *P. aeruginosa *inject toxins from their cytoplasm into eukaryotic target cells through a protein secretory apparatus, the type III secretion system (TTSS)[2]. Activation of this system (especially toxins production and secretion) is dependant on the contact between the bacteria and the host cells *in vivo *and could be triggered by calcium depletion of the growth medium *in vitro*. TTSS is encoded by three classes of genes coding the secretion apparatus, the toxins and the regulators. Four TTSS toxins are known in *P. aeruginosa *to be secreted through the TTTS: ExoS, ExoT, ExoY and ExoU. All three classes of TTSS genes are co-ordinately controlled by a common transcriptional activator ExsA encoded by the *exsCEBA *operon, which controls its own synthesis[3]. The first gene of the TTSS *exsD-pscBL *operon encodes ExsD which inhibits ExsA activity by forming an inactive complex[4]. Fixation of ExsD to ExsA is prevented by ExsC, encoded by the first gene of the *exsCEBA *operon, which interacts with ExsD[5,6]. Finally, ExsE, encoded in the *exsCEBA *operon interacts with ExsC and prevents its binding to ExsD. Upon TTSS activation by calcium depletion, ExsE is secreted through it, thus releasing ExsC and the inhibition of ExsA by ExsD[6,7]. This complex regulatory network is described in figure 1A. However, numerous strains possessing the entire set of genes required for type III cytotoxicity cannot be induced by host contact or calcium depletion unless submitted to *exsA *overexpression[8,9]. These strains will be named non inducible for type III cytotoxicity to distinguish them from truly non cytotoxic ones, such as mutants in TTSS. It has recently been shown that they accumulate in the lungs of cystic fibrosis patients during long term infection.

Epigenetic phenotypic modifications arise and can be transmitted from a cell to its progeny in the absence of any genetic modifications. As a consequence, several phenotypes may arise from the same genome in the same conditions, which is equivalent to the existence of multiple steady states according to physicist's terminology. Such a phenomenon has been under investigations for example in the lactose metabolic network of *Escherichia coli *since 1957 [10-12]. Since all the genes necessary for cytotoxic secretion are present and can be activated in non inducible *P. aeruginosa *strains, this is consistent with the hypothesis that the transition from inducibility to non inducibility of type III cytotoxicity is an epigenetic switch. Thomas conjectured that a positive feedback circuit (comprising interacting elements, each of which exerts, directly or indirectly, a positive action on itself) in a non linear dynamical system, such as a regulatory network controlling a biological process, is a necessary condition (although not sufficient) for the existence of multiple steady states[13,14]. This hypothesis has now been formally demonstrated[15,16]. Thus, the presence of the positive feedback circuit in the TTSS regulatory network where ExsA positively regulates it's own synthesis, could lead to a bistable behaviour for the gene *exsA *(either on or off) corresponding to an epigenetic switch of the TTSS (inducible or not)[17]. However, at least two intertwined feedback circuits control the production of ExsA: a positive feedback circuit at the transcriptional level (ExsA is required for its own synthesis) and a negative feedback circuit (ExsA is required for the synthesis of its inhibitor) (Figure 1B). The existence of the positive circuit is a necessary, not sufficient, condition for multistationarity and more particularly epigenetic bistable switches[13]. Consequently, one has to check if the system actually displays multistationarity. Molecular techniques can not by themselves prove or refute this hypothesis. Indeed, the absence of a mutation can be very difficult to demonstrate, since all possible genes in which a mutation could induce such a phenotypic change may not be known. Furthermore, the presence of a mutation in a putatively involved gene in inducible strains may not mean that it is the initial cause of the phenotypic change: an epigenetic switch could be the first event, followed by a mutation that may further stabilize it. Mathematical analysis of the network's dynamic is therefore necessary to determine if the epigenetic hypothesis is coherent: this means if a reasonable model, which exhibits two stable solutions, can be drawn. As the parameters of the system are not known, we used generalized logics, a method derived from a Boolean approach[18,19].

The actual regulatory network to analyse is the one in figure 1A. However, proteins ExsC and ExsE are modulators of protein ExsD activity, and can be omitted from the model (Figure 1B) as far as the aim of this model is not to describe the whole regulatory network, but only to address the question of the possibility of an epigenetic switch between inducibility and non inducibility of the TTSS. Figure 1C shows the graph that models the minimal regulatory network: vertices represent the biological entities (x = ExsA, and y = ExsD) and lines their interactions ("-" for negative action and "+" for positive action). The output (z) is the production of the secretory devices and toxins secretion under induction by calcium depletion, i.e. inducibility. This graph is similar to the one worked out for the regulation of mucoidy in the same bacteria[20]. This model, a formal computer science approach, using model checking Computation Tree Logic (CTL) and a dedicated framework SMBioNet[21], allowed us to fully establish the consistency of the epigenetic hypothesis for the acquisition of a type III cytotoxicity and to propose experimental evidences that are sufficient to prove it. We thus could design *in vivo *and *in vitro *experiments to test it.

## Results

### Computer modelling

#### Generalised logical analysis

**Figure 2:**
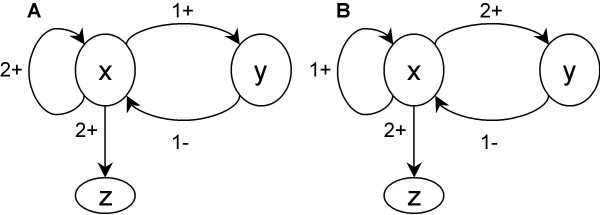
**Underlying dynamics depend on the values of interactions thresholds and on the parameters values**. The labels of the vertices indicate the sign of the interaction and its threshold. As these thresholds are almost always unknown, value 1 means only that the corresponding threshold is the lowest one, and value 2 that it is the second lowest threshold. Two different graphs must then be drawn depending on which promoter is more sensitive to ExsA. (A) In this case, the threshold above which x is active on x is higher (level 2) than that above which it is active on y (level 1). (B) Represents the reverse case. z is an output element whose level is determined by the value of x: low values of x will lead to negligible amounts of z, while high values of x will lead to high levels of z.

Logical analysis relies on a simplification of the sigmoid curve representing the action of a transcriptional activator as a step function, defining a threshold of concentration above which the activation takes place[19]. In the case of the network shown in figure 1C, x has two distinct actions and it is very unlikely that the concentration thresholds above which protein ExsA is active on the promoter of gene *exsD *and on the promoter of its own gene are identical. Consequently, we associate two threshold values with x and treat it as a three-level logical variable (0, 1, 2). Variable y has one action only and is treated as a two-level logical variable (0, 1). Therefore, two different graphs must be drawn depending on which promoter is the most sensitive to ExsA (Figure 2). For simplicity, we consider only the two master elements, x and y.

Another aspect of the generalised logical method is that it allows analysis of the dynamic properties of a network in terms of the "functionality" of its constitutive feedback circuits. The effect of a circuit does not only depend on the mere existence of the relevant interactions, but also on their relative strengths, which are noted as discrete parameters (K). For a given regulatory network, the number of dynamics depends on the number of thresholds and parameters[17]. In the case of the two graphs depicted in figure 2, there are 324 different combinations of parameters for each graph. Each set of parameters defines a specific temporal behaviour. Different set of values of the parameters can lead or not to epigenetic behaviour.

#### The use of a formal computer science method allows to select parameter set that lead to a model exhibiting the hypothesised behaviour

Finding suitable valuations for parameters constitutes a major issue for the modelling. We runned the whole corpus of formal methods from computer science to include the dynamical knowledge or hypotheses on the system[21]. Our method starts by expressing the biological hypothesis (i.e. the epigenetic behaviour) formally, as formal sentences which can be manipulated automatically by computer. Here we applied a widely used temporal logic called Computation Tree Logic, because time plays a central role in behavioural properties. Moreover, formal sentences in CTL can be automatically checked against the 648 models by using a Model Checking algorithm. Technically, behaviour is represented by a transition system (state graph), which can be automatically computed from the regulatory network and the values of parameters. Then, it becomes possible to extract (*via *the 'brut force technique') the sets of parameters, if any, which lead to a behaviour that satisfies the CTL sentences. Model Checking thereby classifies the set of possible dynamic behaviours into two groups: those which satisfy the property and those which do not. This methodology is instrumented by the software SMBioNet [21] which, for the given regulatory network, automatically returns all sets of parameters which make the hypothesis coherent with the model.

**Figure 3:**
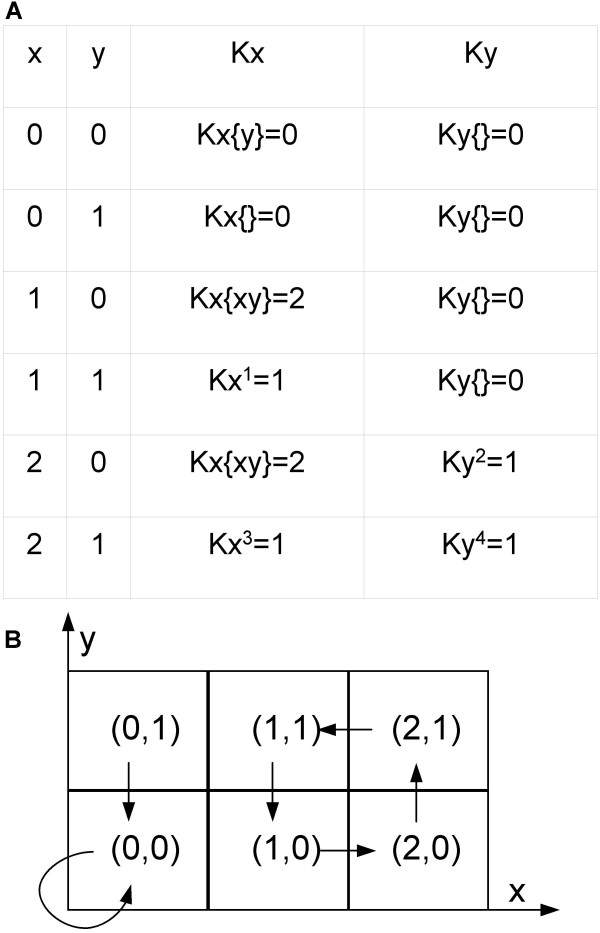
**One result from SMBioNet in accordance with the epigenetic hypothesis of the regulatory graph**. SMBioNet provides a graphical interface that allows the user to define a regulatory graph and to edit temporal properties. SMBioNet exhibits all sets of parameters which satisfy the properties. Consistency is thus established if and only if at least one set of parameters is selected by SMBioNet. (A) Parameter table. The two first columns list all possible states of the network according to genes x and y. The third column gives the Kx, w parameters which define the expression level towards which x tends to evolve. Note that w represents all the positive regulatory effects that are active on x (including the lack of active negative effects). Here, w reflects the effects of x, if its value is higher than the activity thresholds indicated in the graph, and the effects of y, if its value is lower than the activity threshold. The fourth column similarly gives the Ky, w parameters which define the expression level towards which y tends to evolve. (B) State transition graph. The dynamics of the model are deduced from the values of the parameters *via *a desynchronisation algorithm[21].

In the present case, the dynamical hypothesis (epigenetic hypothesis) can be translated into two CTL. In the first, the non inducible phenotype is stable in most of the bacteria. The sets of parameters to be considered for consistency must therefore induce a behaviour where a non-cytotoxic bacterium (z = 0) cannot subsequently become cytotoxic, in the same conditions. This is formally expressed in temporal logic as: (z = 0) = > always (z = 0). In the second CTL, the cytotoxic phenotype is stable. Thus, even in the presence of the inhibitor (y) there is a state in which inducibility of the type III cytotoxicity (z = 1) is activated recurrently (i.e. z is activated in a recurrent way even if the y♦ x inhibition is functionally active). This means that if at a given time the bacterium has acquired the new phenotype, then later on, it will be again in the same state. This is formally expressed in temporal logic as: (z = 1) = > Fs (z = 1), where Fs means "in a strict future". The epigenetic hypothesis is consistent, if and only if, at least one set of parameters leads to a behaviour satisfying these two formal properties. From figure 2, z = 1 requires that x = 2. Consequently the epigenetic hypothesis means that it is possible to make recurrent (x = 2) and the previous formulae are equivalent to [((x = 2) = > Fs(x = 2)) and ((x = 0) = > always(not(x = 2)))] as well as to [((x = 2) = > Fs(z = 1)) and ((x = 0) = > always(z = 0))]. SMBioNet formally proves that the epigenetic hypothesis is consistent because 8 models satisfy these formulae, 2 of which are in agreement with the known biological facts. The state graph, for one of these 2 models, is displayed in figure 3.

#### Design of a discriminating experimental scenario

In addition to proving the consistency of the epigenetic hypothesis, formal methods can establish the discriminating power of the experimental scenario. The goal is to prove both formal sentences *in vivo*. The two previous formulae reflect the existence of two steady states. As indicated above, the first formula states that the basal level of x is a steady state and is consequently satisfied *in vivo *which is true for a non inducible strain. Thus, only the second formula remains to be experimentally validated. The second formal sentence ((x = 2) = > Fs (z = 1)) is always true when x ≠ 2 because, according to the truth table, the implication (false = > anything) is always true. Consequently it is unnecessary to conduct an experiment where x ≠ 2. Therefore any experiment must start by pushing the concentration of x to 2 which means to pulse x to saturation with an external signal. Moreover Fs(z = 1) signifies "wait a lapse of time to allow the system to settle down and check if the bacterium has acquired a cytotoxic phenotype". If the bacterium has not changed its phenotype, then the experiment *a priori *fails. Consequently the second part of the experimental scenario is rigorously sufficient to check the second formal sentence. The length of this "lapse of time" must be determined experimentally; here it stands for "as many generations later as possible". If the bacterium has acquired an inducible phenotype, that lasts at least several generations after the external signal has been removed, then epigenesis is proven.

This formal approach was useful to suggest *in vitro *and *in vivo *experiments to test our hypothesis. It showed that the two stable attractors differ only by the amount of ExsA protein, and that, if the hypothesis is correct, no differences in the concentration of the inhibitor ExsD can lead to a change in phenotype (Figure 3B). Thus, if a transient increase in the amount of ExsA protein can shift the system from a non cytotoxic to a stable cytotoxic state, the existence of the epigenetic switch would be demonstrated.

### Experimental results

#### An ExsA pulse allows to recover a stable type III secretory phenotype

Experiments suggested by the formal method require pulsing ExsA by application of an external stimulus and observing the change in phenotype (if any) and its stability when the stimulus is removed. In order to pulse ExsA, we added an additional *exsA *gene, under the control of an inducible promoter, to a non inducible *P. aeruginosa *strain PAO1. Thus, we constructed plasmid p*exsA*ind. In this construction, transcription of *exsA *is under the control of the p*tac *promoter repressed by the LacI protein produced in large amounts because of the presence of the *lacI*^*q *^gene[22]. Under inducing conditions (presence of isopropyl-beta-D-thiogalactopyranoside, IPTG), transcriptional repression by LacI is inhibited, permitting over-expression of the *exsA *gene from the p*tac *promoter. This induction is immediately released when the medium is depleted of the inducer. The most straightforward test to determine the resulting phenotype is the electrophoretic detection of the type III toxins secreted *in vitro *after bacteria have been submitted to calcium depletion.

**Figure 4:**
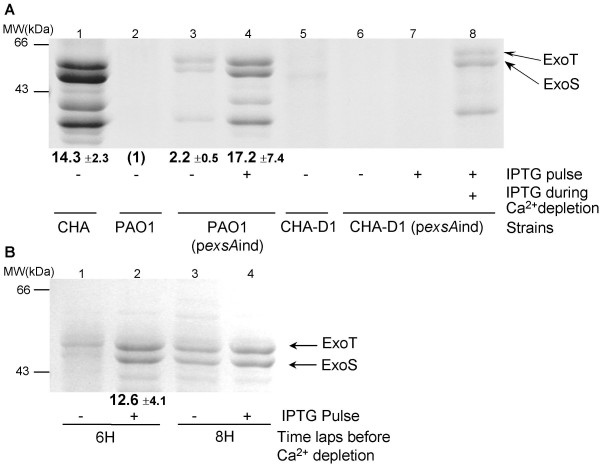
**Secretion profiles of various *P. aeruginosa *strains induced for type III secretion**. Coomassie blue-stained SDS-PAGE of TTSS secreted proteins. ExoS and ExoT toxins, the most abundant type III-related exoproducts[3], are indicated on the right side of the gel. IPTG was used at 1 mM. Under corresponding lanes are given the mean values (± SE) of the relative *exsA *mRNA production compared with the one of PAO1 (set to 1), measured by real time PCR in the same conditions. (A) Lane 1, CHA; lane 2, PAO1; lanes 3 and 4, PAO1 (p*exsA*ind) without or with a 20 min IPTG pulse; lane 5, PAO1 ExsA^-^; lanes 6 and 7, PAO1 ExsA^- ^(p*exsA*ind) without or with a 20-min IPTG pulse; lane 8, PAO1 ExsA^- ^(p*exsA*ind) with a 20 min IPTG pulse and with IPTG during the calcium depletion. (B) PAO1 (p*exsA*ind) was pulsed or not with 1 mM IPTG during 20 min then washed with fresh LB. Next, bacteria were maintained in exponential phase of growth by serial dilution with fresh medium during a definite time before calcium depletion as indicated below the gel.

Strain PAO1 used in this study was described as unable to induce death of human polymorphonuclear neutrophils through the TTSS[23], and is non inducible (does not produce type III toxins). This strain was transformed with p*exsA*ind. In the absence of inducer it produced no, or very little, toxin after 3 hours of culture in calcium-depleted medium (Figure 4A, lane 3). Strain CHA, a cytotoxic strain that secretes large amounts of toxins ExoS and ExoT[23], was used as a positive control (Figure 4A, lane 1). The presence of IPTG during the 3 h of growth (not shown) or for a 20 min pulse before the 3 h culture (Figure 4A, lane 4) results in substantial toxin production. We observed this epigenetic switch from a culture grown up to 6 hours (about 7 generations), in Luria Bertani medium (LB) without EGTA after the ExsA pulse, before the leakiness of the construct became a drawback (Figure 4B, lane 1 *vs*. 3). The secretory phenotype, under calcium depletion, acquired by PAO1 (p*exsA*ind) is therefore stable for about 10 generations, after the pulse of ExsA production from the additional gene on p*exsA*ind. In contrast, a CHA ExsA^- ^mutant (CHA-D1) transformed with p*exsA*ind did not produce toxins in absence of IPTG nor after a 20 min pulse (Figure 4A, lane 6–7). Secretion of type III toxins from this strain was only obtained when IPTG was present in the medium all along the calcium depletion (Figure 4A, lane 8). This shows that induction of toxin secretion by a 20 min pulse in strain PAO1 required the presence of an active feedback loop on *exsA*. Transient production of ExsA is not able to promote inducibility in the absence of an active *exsA *gene under the regulation of its own autoactivated promoter. It also shows that this result was not an artefact. Indeed, leakiness of the construct or noise, high production of protein ExsA upon a 20 min IPTG pulse (the *exsA *gene is on a high-copy-number plasmid and under the control of a strong promoter), or even persistence of some IPTG in the culture medium after 3 h of growth cannot be responsible for toxin secretion. Whatever the remaining production or presence of ExsA protein in the bacteria after four generations without IPTG, it is not high enough to promote toxin secretion by itself. To confirm that TTSS inducibility observed was correlated to ExsA expression we determined relative *exsA *mRNA level using real time PCR under the same experimental conditions used above. Results are indicated under corresponding lanes in figures 4A and 4B. Relative expression levels of *exsA *in strains CHA, PAO1 (p*exsA*ind) none pulsed or pulsed were respectively 14.3 ± 2.3, 2.2 ± 0.5 and 17.2 ± 7.4 times higher than in strain PAO1 (Figure 4A). In addition, after 10 generations following IPTG pulse, *exsA *level in strain PAO1 (p*exsA*ind) was still 12.6 ± 4.1 times higher than in strain PAO1 under calcium depletion (Figure 4B). These results can be compared to that obtained using toxins secreted through the TTSS in the same experimental conditions and show that the level of secreted toxins is correlated to *exsA *expression in these conditions.

#### PAO1 (pexs*A*ind) acquires an *in vivo *type III cytotoxicity after a transient increase of ExsA

**Figure 5:**
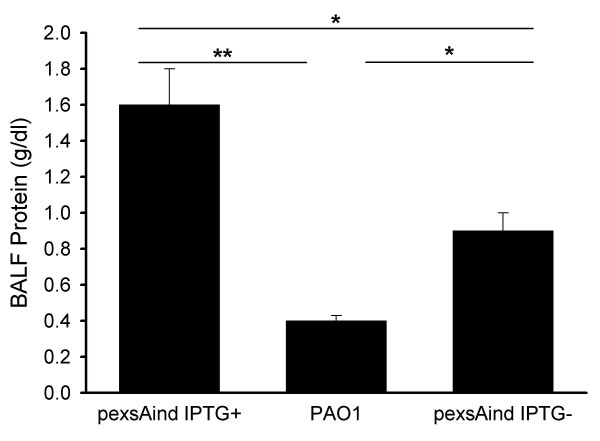
**Epigenetic acquisition of *in vivo *type III cytotoxicity**. Protein levels measured in the bronchoalveolar lavage fluid (BALF) in the three experimental groups: PAO1 pexsAind (10^8 ^CFU/ml) with 1 mM IPTG Pulse (pexsAind ITPG+) or not (pexsAind IPTG-), PAO1 (10^8 ^CFU/ml). Proteins levels are significantly different between each group. *p < 0.01, **p < 0.0001. Results are expressed as mean ± SE.

A clinical correlation between type III secretory protein phenotype and lung injury severity has previously been shown[24,25]. Indeed, unlike the wild type strain CHA, an *exsA *null mutant induces no increase of protein concentration in the bronchoalveolar lavage fluid (BALF) during the infection[26]. Therefore, we examined, through an acute pulmonary infection model, the cytotoxicity of PAO1 carrying or not p*exsA*ind. Strains were pulsed or not with IPTG during 20 min, washed and then cultivated in tryptic soy broth before infection. The injury of the alveolar capillary barrier was estimated by the amount of proteins recovered in the BALF (Figure 5). A bacterial inoculum of 10^8 ^CFU/ml, strain PAO1 (p*exsA*ind) treated with an IPTG pulse, led to a higher quantity of proteins (1.6 ± 0.2 g/dL) compared to the wild type strain (0.4 ± 0.03 g/dL) even if the latter was injected at a ten times higher inoculum (1.04 ± 0.21 g/dL for PAO1 at 2.10^9 ^CFU/ml, data not shown). The non pulsed PAO1 (p*exsA*ind) strain showed protein level ranging between the pulsed and the wild type strain which was significantly different. But this strain showed no significant difference with the wild type strain injected at a ten times higher inoculum. Although results obtained with the non pulsed strain PAO1 (p*exsA*ind) remained unclear, this experiment illustrated the stable acquisition of cytotoxicity by a transient increase of the main transcriptional regulator, ExsA.

## Discussion

Despite a functional wild-type genomic background, some *P. aeruginosa *strains are unable to develop a type III dependent cytotoxicity except with an over-expression, *in trans*, of the transcriptional activator of this virulence system, ExsA[8]. We proposed to call these strains non-inducible for type III cytotoxicity. This observation and the presence of a positive feedback loop regulating the expression of *exsA *led us to hypothesize the possibility of an epigenetic switch between inducibility/non inducibility of the type III dependant cytotoxicity in *P. aeruginosa*. With the help of generalised logic and a formal computer approach we designed a minimal model of *P. aeruginosa *type III basic regulation under calcium depletion and established the consistency of the hypothesis of an epigenetic acquisition of an inducible type III cytotoxicity. This model also showed that in this case, and in this case only, an artificial (exogenous) transient increase in protein ExsA would suffice to permanently switch a non inducible strain to inducibility. Next, we engineered an *exsA *inducible gene. With this construction, we demonstrated that a stable acquisition of the secretory phenotype after a transient signal relies on the presence of the feedback loop of the auto regulated *exsA *gene in agreement with the model. These non inducible strains thus switched to inducibility, also showed the acquisition of *in vivo *inducible type III cytotoxicity. Since the *exsA *gene added *in trans *was under the control of the inducible promoter, p*tac*, we have experienced leakiness or noise. This phenomenon, ubiquitous in biological systems, is responsible for cell-to-cell variation in gene expression[27]. It has been shown to be the cause of population bimodal heterogeneity, when coupled with a feedback circuit responsible for bistability, for instance in the repartition of lytic/temperate phages in the population upon infection of *E. coli *by bacteriophage-λ [28]. In some individuals in the population bearing pe*xsA*ind such a noise-dependant switch could be responsible for the slight activity observed with non induced PAO1(p*exsA*ind) strain in *in vitro *experiments (Figure 4B, lane 3) and the *in vivo *increase of cytotoxicity of this strain. Moreover it has been shown *in vitro *that metabolic state, which is probably modified in lungs, regulates the percentage of cells able to induce type III secretion gene expression under inducing conditions evidencing that only a subpopulation could induce type III gene expression under inducing conditions[29]. Although this does not prove that this is the mechanism involved to acquire or lose type III cytotoxicity during infection, the results obtained *in vivo *make the epigenetic hypothesis very likely. However, this hypothesis does not necessarily apply to the cytotoxicity of other bacteria, nor to mucoidy in *P. aeruginosa *for which an epigenetic acquisition had been proposed[20]. In each case, the hypothesis must be tested, first by modelling then experimentally.

## Conclusion

These *in vitro *and *in vivo *experiments, in accordance to the predictions of the formal method, indicate that a stable acquisition (or loss) of a phenotypic trait involved in the pathogenicity of *P. aeruginosa *can arise from an epigenetic switch. Direct therapeutic consequences could arise for *P. aeruginosa *infections especially in cystic fibrosis. Any therapy inhibiting the type III regulon would reduce the pathogenicity of the bacteria and the severity of the infection. Since the inducibility of type III secretory phenotype is a bistable phenotype, a reduction of ExsA activity below the triggering threshold would be sufficient to impede the positive feedback circuit leading to reversion of the type III secretory phenotype and reduction of the pathogenicity of the bacteria. To do so, we may investigate the use of quorum sensing signals as the Rhl quorum sensing system represses TTSS expression[30,31] or the effect of small molecules able to block TTSS toxins secretion as shown in *Yersinia *which could lead to a repression of the system due to the absence of the secretion of the inhibitor ExsE[32]. According to the hypothesis that planctonic type III secreting form of *P. aeruginosa *is responsible for early and invasive phases of the infection [33-35], such a therapy would improve antibiotic performance and other treatments to eradicate the infection or limit spreading.

## Methods

### Bacterial strains and growth conditions

*Pseudomonas aeruginosa *strains used in this study were: CHA, a cytotoxic isolate from a cystic fibrosis patient[36] which secretes toxins under inducing condition for the type III secretion system[23]; PAO1, a noncytotoxic strain widely used in laboratory studies; PAO1 ExsA^-^, an isogenic mutant of PAO1 unable to synthesize ExsA. PAO1 ExsA^- ^was obtained using a mutation method based on *sacB *negative selection and cre-lox antibiotic marker recycling[37]. Strains were routinely grown in Luria-Bertani broth (LB), Tryptic Soy Broth (TSB) or plated on PIA (Pseudomonas Isolation Agar, Difco, France). The p*exsA*ind plasmid was introduced into PAO1 and its isogenic mutant by electroporation and maintained with 300 μg of carbenicilin/ml.

### Construction of an inducible exsA gene

The *exsA *gene was amplified by PCR using the high fidelity polymerase PfuUltra (Stratagene) with *P. aeruginosa *chromosomal DNA as the template and primers EXSAS and EXSAR (see table 1). Their 5' termini contain *EcoR*I and *Hind*III restriction sites, respectively. PCR products were ligated into pCR^®^-Blunt II-TOPO^® ^vector (Invitrogen). The *EcoR*I-*Hind*III *exsA *fragment from this plasmid was inserted into pTTQ18[22], digested by the same enzymes. This inducible system was amplified from pTTQ18*exsA *with the Xbaptac and KpnlacIq primers (see table 1), carrying *Xba*I and *Kpn*I restriction sites, respectively, and inserted into the vector pCR^®^-Blunt II-TOPO^®^. For the final construct, p*exsA*ind, the *Xba*I-*Kpn*I fragment from the previous vector containing *exsA *was ligated into pUCP20[38], a high-copy-number shuttle vector for *P. aeruginosa*.

### Reverse transcription

Total RNA were extracted after calcium depletion using HighPure RNA Extraction kit (Roche) according to the manufacturer's instructions. Reverse transcription was realized using the Transcriptor reverse transcriptase (Roche) with 1,33 μg total RNA, 1 mM dNTP and 1 mM of primer 16sas or RTExsA (Table 1). Primer and RNA were mixed, heated for 10 min at 65°C then dNTP, buffer and enzyme were added. cDNA were obtained after a reaction conducted for 30 min at 55°C, followed by 5 min at 85°C.

### Real time PCR

Real-time PCR assays with hydrolysis probes were conducted on a LightCycler apparatus (Roche), using FastStart DNA Master Hybridization Probe kit (Roche) by following the manufacturer's instructions. Sequences of *exsA *gene and *16s rRNA *gene used has housekeeping gene were obtained from the *P. aeruginosa *genome sequence from Pseudomonas Genome Project[39]. Primers and probes (table 1) were designed and obtained from Proligo^® ^(France) and were used at final concentrations of 0.75 μM and 0.2 μM, respectively. Final MgCl_2 _concentration was adjusted to 4 mM. Volume of cDNA from reverse transcription was 5 μl per assay. Thermal cycling conditions were 10 min at 95°C, followed by 45 cycles of 10 s at 95°C and 10 s at 58°C. Relative expression level of the *exsA gene *to the *16s rRNA *gene was calculated using the Lightcycler software 4.0 for each strain and reported to *exsA *relative expression of the strain PAO1. Values are the mean (± standard error) of three experiments.

### SDS PAGE

Bacteria were cultivated in LB broth overnight, diluted to 1.2 × 10^8^cfu/ml in LB supplemented or not supplemented with 1 mM ITPG and incubated with aeration for 20 min at 37°C. Then, bacteria were spun down, washed twice in LB and grown with aeration for 3 h at 37°C in the presence or absence of 1 mM IPTG in conditions of calcium depletion: LB medium supplemented with 5 mM EGTA and 20 mM MgCl_2 _(inducing conditions known to induce secretion of type III toxins *in vitro*[40]). Bacterial densities were determined by optical density measurement at 600 nm. Cultures were spun down, and proteins in the supernatant were precipitated by perchloric acid (precipitated volume was normalised to 9 × 10^8 ^cfu) and washed with acetone. Proteins were separated on a 12% SDS-PAGE, and stained with Coomassie blue. In the secretion experiment with a delay between the IPTG pulse and calcium depletion (Figure 4B), bacteria were pulsed with 1 mM ITPG for 20 min then washed with LB. Next, they were maintained in exponential phase of growth by serial dilution with fresh medium during a definite time before calcium depletion.

### Animals and infection model

Specific pathogen-free Sprague Dawley rats (n = 40) (280–320 g), (Charles River Laboratoires France, St Germain/l'Arbresle, France) were housed in the Lille University Animal Care Facility and allowed food and water *ad lib*. All experiments were performed with approval of the Lille Institutional Animal Care and Use Committee. *P. aeruginosa *strains were cultivated in Triptic Soy Broth (TSB) broth overnight, diluted to 1.2 × 10^8^cfu/ml in TSB supplemented or not supplemented with 1 mM ITPG and incubated with aeration for 20 min at 37°C. Cultures were then centrifuged, washed twice and grown with aeration at 37°C in tryptic soy broth medium. After 3 hours, bacteria were washed and resuspended in physiological serum to reach a final concentration of 10^8 ^or 2 × 10^9 ^cfu/ml evaluated by spectrophotometry. Acute lung injury was produced according to the method described by Pennington and Ehrie[41]. Under short anaesthesia, a small midline incision was made on the neck ventral surface after swabbing it with ethanol. The trachea was exposed by blunt dissection. Using a 28-gauge needle, 0.5 ml/kg of bacterial suspension was instilled into the trachea, followed by injection of 0.5 ml of air[42,43]. The animals were studied 24 h after instillation of the bacteria.

### Bronchoalveolar lavage (BAL)

Bronchoalveolar lavage was performed by cannulating the trachea. Lungs from each experimental group were washed with a total of 20 ml in 5-ml aliquots of PBS with 3 mM EDTA. The returned fluid was pooled and centrifuged (200 g for 10 min). BAL fluid (BALF) samples were filtered and immediately frozen at -80°C after collection. Protein concentration in the BALF was measured with an automated analyzer (Hitachi 917, Japan).

## Authors' contributions

DF participated to the design of the study, carried out the microbiological and animal experiments and drafted the manuscript. RL carried out animal experiments. BG carried out animal experiments and drafted the manuscript. JFGM proposed the model while GB and JPC designed and performed computer studies and draft the manuscript. AM, BP, JFGM conceived the study and participated to its design and coordination and drafted the manuscript. All authors read and approved the final manuscript.

## Figures and Tables

**Table 1:** PCR primers and probes used, restriction sites are in bold (6-FAM, 6-carboxyfluorescein; TAMRA, 6-carboxytetramethylrhodamine).

Name	Sequences (5'-3')	Description
EXSAS	CC**GAATTC**TTATAATATGCAAGGAG	Amplification of *exsA*
EXSAR	GG**AAGCTT**TCAAAAAACGTCAGTTA	
Xbaptaq	CG**TCTAGA**TTGACAATTCATCGCCTCG	Amplification of *exsAind*
KpnlacIq	G**GGTACC**TCACTGCCCGC TTTCCAGT	
RTExsA	CAGCGAACGCGATGATGCC	Reverse transcription of *exsA*
ExsAQS	GAACTGACCGTCCAGGACAT	Amplification and detection of *exsA *cDNA in RT PCR
ExsAQR	CAACGCTCGACTTCACTCAA	
ExsADL	(6-Fam)CCTGGCGAGTTGCTTTTCGTCCGC(Tamra)	
16s sens	AAGCAACGCGAAGAACCTTA	Amplification and detection of *16S rRNA *cDNA in RT PCR
16as	CACCGGCAGTCTCCTTAGAG	
16sDL	(6-Fam)ACGAAGGGACGCACGAG(Tamra)	
